# Antenatal care visit attendance, intermittent preventive treatment and bed net use during pregnancy in Gabon

**DOI:** 10.1186/1471-2393-13-52

**Published:** 2013-02-26

**Authors:** Marielle Karine Bouyou-Akotet, Denise Patricia Mawili-Mboumba, Maryvonne Kombila

**Affiliations:** 1 Department of Parasitology-Mycology, Faculty of Medicine, Université des Sciences de la Santé, Libreville, BP 4009, Gabon; 2 Malaria Clinical and Operational Research Unit (MCORU), Centre Hospitalier Regional de l’Estuaire Melen, Libreville, Gabon

## Abstract

**Background:**

The World Health Organization (WHO) recommends that intermittent preventive treatment with sulfadoxine-pyrimethamine (IPTp-SP) and insecticide treated bed nets (ITNs) must be provided during antenatal care (ANC) visits for malaria prevention during pregnancy. The aim of this study was to determine the level of ANC attendance and its relationship with IPTp-SP and bed net coverage in Gabonese pregnant women.

**Methods:**

This was a cross-sectional survey performed in 2011 in sentinel sites for malaria: two ANC units (Melen and Owendo) and one delivery unit (CHL). A validated structured questionnaire was used to collect the following data: age, parity, history of the current pregnancy including gestational age at the interview, number of ANC visits already performed, date of first visit, use of malaria preventive measure and details on IPTp-SP administration.

**Results:**

During the study, 1030 women were interviewed, 735 at their ANC visit and 295 at the delivery. Their median age was 24[20–29] years and 21.0% were primigravidae. More than 70.0% attended their first ANC visit during the second trimester. Among the 442 women who were at the end of their pregnancy, 71.5% had a correct attendance, at least four ANC visits, most frequently women with no education and older women; IPTp-SP was offered to 84.1% of them and 57.4% received at least two doses. The number of SP doses was correlated to the number of ANC visits. Bed net coverage was 59.0%, not associated with ANC attendance. Among the women with correct ANC attendance, only 49.5% had a complete IPTp-SP course associated with bed net use during pregnancy. In the site where SP administration was supervised, 80% had four ANC visits and 97.4% received a full 2-dose course of IPTp-SP.

**Conclusions:**

Despite a high level of correct ANC attendance in Gabon, the goal of 80% of women with 2-dose IPTp-SP during pregnancy is not achieved. Evaluations, training of health workers, as well as surveys from other areas of the country are needed to further measure the implementation and the impact of these strategies.

## Background

In 2007, about 32 million women were pregnant in malaria endemic areas in Sub-Saharan Africa [1]. The World Health Organization (WHO) guidelines for malaria prevention during pregnancy include use of intermittent preventive treatment with sulfadoxine-pyrimethamine (IPTp-SP), sleeping under an insecticide treated bed net (ITN) and prompt management of malaria cases and anaemia [2]. These strategies have been adopted by 35 of the 45 African endemic countries including Gabon, and should be delivered through collaboration between reproductive health systems and malaria control programs, during the four target antenatal care (ANC) visits throughout the pregnancy as recommended by WHO [2-5]. Although a high proportion of pregnant women had an ANC visit at least once during pregnancy, coverage level of IPTp-SP and ITNs remains low in Sub-Saharan Africa [6-8]. In 2010, it was estimated that 55% of pregnant women received a full IPTp-SP dose (at least two curative doses) during ANC visits [3]. Few studies have analyzed the relationship between the number of ANC visit, IPTp-SP and bed net coverage, the reported data are controversial [8,9]. In Gabon, during the last ten years, more than 70% of women have attended ANC at least twice during their pregnancy, correct IPTP-SP and bed net coverage were estimated at around 56% and 50% in 2006, respectively [10]. Awareness campaigns in recent years for both health workers and pregnant women were organized by the Gabonese Malaria National Control Program (MNCP) supported by the Global Fund. An evaluation of the implemented programs is needed, including the key indicators of intervention coverage, ANC visit planning and attendance, health workers adherence to the recommendations and local socio-demographic and economic factors [11-13]. We present in this study the quality of care for pregnant women through ANC attendance and its relationship with access to adequate malaria preventive measures in Libreville.

## Methods

Malaria transmission is perennial in Gabon with slight seasonal fluctuations. The annual entomological inoculation rate is estimated at 33.9 infected bites per person per year, *Anopheles gambiae s.s* is the predominant vector [14]. *Plasmodium (P.)falciparum* infection prevalence is respectively of 24%, 31% and 11% at Libreville, Melen and Owendo.

### Study sites

MNCP has five sentinel sites (represented by one public hospital in five provinces) for malaria survey and management across the country. This baseline survey took place in the Centre Hospitalier de Libreville (CHL) and the Regional Hospital of Melen (RHM), two sentinel sites located in and around the capital city of Libreville, where more than 45% of the Gabonese population resides; and at the public reproductive health centre at Owendo (HCO), a suburban area located at 25 kilometers from the capital city. According to the national reproductive health program organization, women have access to ANC visits, ITNs and IPTp in public health centers of the city.

### Study type and population

This study is a cross-sectional and observational survey conducted from March 2011 to September 2011. Antenatal care and delivery units were surveyed during a 10 week- period following a two to three week planning and discussion with health centre teams. To assess the burden of malaria during pregnancy, a structured questionnaire adapted from the framework proposed by the CDC, was prepared by the team of the Department of Parasitology at the Faculty of Medicine, University des Sciences de la Santé in Libreville. All eligible women attending ANC and delivery units were invited to participate and oral consent was required before proceeding. Women were eligible for the survey if they met the following criteria: had experienced quickening (i.e., the recognition of fetal movement) during ANC or were nearing the end of a normal pregnancy; had a normal (non complicated) delivery; and were older than 14 years.

### Administration of the survey

Each women attending the ANC or the delivery unit was interviewed by a field worker and the midwives for 15 minutes. Socio-demographic data including age, residence, language used at home, matrimonial status, education level, and profession of the head of the household were recorded. Antenatal data were obtained from either the antenatal card or the register of the centre with assistance of a midwife and included: parity, gestational age at the interview, number of ANC visits already performed, date of the first visit. Gestational age was determined, after the physical examination by the midwife who used a gestational calendar or uterine growth when the date of last menses was not known. Use of malaria prevention was recorded from both ANC card and the health centre maternal care register. If prevention was performed, the type of medicine taken was recorded as well as the first time of dosing, the number and dates of drug administration, and the number of tablets taken. Women were asked if they sleep under a bed net, if they used it the night before the interview, if the net was treated with insecticide or if it was a long lasting insecticide treated net.

### Sample size estimation

Sample size was calculated by considering the previous data estimating that i) 80% of pregnant women have at least 2 ANC visits, complete ANC attendance is estimated to be between 50% and 70%, ii) IPTp-SP coverage was at 50% in 2006 and will be between 60 to 70% five years later, and iii) each year less than 2000 women are received in each reproductive health service [8,9]. A sample size calculation was done using STATA software and Epi-Info 6 with a “design effect” of 2 (since the present survey is not a random community sample study), a risk of 5% and a 90% power. Therefore a total number of 360 interviewed pregnant women (120 per unit) was estimated.

### Data management and analysis

A full course IPTp-SP was defined as at least a complete 2-dose course of SP administered during the pregnancy. As the investigators worked closely with the midwives during ANC, sites were classified according to the way the SP was given to the women: supervised SP administration when complete dose was given as directly observed by the nurse and unsupervised SP administration when complete dose was given to the women who had to take it at home. Site supervisors checked all questionnaires for completeness every day at the end of all interviews. The log of enrollees and refusals for both the antenatal care clinic and delivery were kept up to date. Two data entry clerks double entered the data using Epi info version 2000 and analyzed with Stata 9.2 (Stata Corporation, College Station, TX USA). Continuous data are presented as medians [25^th^ and 75^th^ percentiles]. Differences between groups were assessed using chi-squared or Fisher’s exact tests for proportions, Student’s *t*-test, analysis of variance (ANOVA) or Kruskal-Wallis test as appropriate. Spearman’s test was used to assess the correlation between continuous variables. The association between some variables was assessed in univariate analysis using odds ratio and the 95% confidence interval (OR (95%CI)). A *p*-value of less than 0.05 was considered significant.

### Ethical consideration

The study was approved by the Gabonese Ministry of Health (GMH). Prompt malaria diagnosis and accurate treatment, drug resistance monitoring and interventions coverage in sentinel sites are the main strategies for malaria control of the GMH represented by the MNCP. The Department of Parasitology-Mycology (DPM) is the reference laboratory for malaria survey including diagnosis, anti-malarial drug resistance evaluation, treatment efficacy and impact of control strategies. The DPM (including the MCORU) is committed by the GMH to carry out these evaluations in collaboration with MNCP throughout the country, in order to provide reliable data for policy adjustment. All data obtained are part of routine activities in sentinel sites and at HCO that are under the administrative supervision of the MH. Women were informed about the study protocol, and their oral consent was required prior to the interview and for data publication.

## Results

### Characteristics of the study population

Overall, 1030 women were interviewed, 735 were seen in ANC units, either at Owendo (n = 219) or Melen (n = 516) and 295 delivered at the CHL. Their median age was 24[20-29] years, the mean gravidity of 3[2-5], 79.0% and 21.8% were multigravidae and teenagers (younger than 20 years old) respectively (Table 1). Pregnant women aged less than 17 years old represented 23.8% (n = 19/80) of the teenagers. The median gestational age was 33[25–38] weeks; the proportion of women in the 2^nd^ trimester was 30.3%. Malaria preventive measures were not used by 96 (9.3%) women and among the 608 (59.0%) who had a bed net at home, 547 (90.0%) used it the night before the interview. A total of 34.0% of the nets used were insecticide-treated.

**Table 1:** Characteristics of all interviewed pregnant women

	**All women (n = 1030)**	**Term and delivery (n = 442)**
Age*		
- Median [IQR]	24 [20–29]	26 [21–30]
- <20 years, n(%)	224 (21.8)	80 (18.1)
- 20–25 years, n (%)	292 (28.4)	123 (27.8)
- >25 years, n (%)	512 (49.8)	239 (54.1)
**Primigravidae, n (%)**	216 (21.0)	85 (19.2)
**Bednet use, n (%)**	608 (59.0)	261 (59.0)
**IPTp-SP, n (%)**	830 (80.6)	372 (84.1)
**IPTp-SP 1**^**rst **^**dose, n (%)**		
1^rst^ trimester	49 (5.9)	
2^nd^ trimester	658 (79.3)	
3^rd^ trimester	123 (14.8)	
**First ANC visit, n (%)**		
1^rst^ trimester	225 (21.8)	
2^nd^ trimester	766 (74.4)	
3^rd^ trimester	39 (3.8)	

* : age unknown for two women.

### Antenatal care attendance

The majority of women (n = 766) attended their first visit during the 2^nd^ trimester (Table 1).

Data from women who completed their last ANC visit and who delivered at the CHL (n = 442) were used to examine the association between ANC attendance, use of bed nets and IPTp-SP. This group differed from the overall population: they were older (median age, 26 *versus* 24 years) and had a lower proportion of young women (*p <* 0.01). Only 3.4% (n = 15) had one ANC visit and 71.5% had at least four ANC visits during the present pregnancy. Median age was the highest in the group of women with complete ANC attendance (considered to be at least four ANC visits during pregnancy); and 15.2% of them had higher educational status, this proportion was three fold lower in the group of those with an incomplete attendance (Table 2). A trend towards a better ANC attendance was also observed in primigravidae women (Table 2).

**Table 2:** Relationship between ANC visits, individual factors and use of preventive measures

	**≤ 3 ANC visits (n = 126)**	**≥ 4 ANC visits (n = 316)**
**Age in years, median [IQR]**	24 [20–29]	26 [21–30]
- <20 years, n(%)	29 (23.0)	51 (16.1)
- 20–25 years, n (%)	43 (34.1)	80 (25.3)
- >25 years, n (%)	54 (42.9)	185 (58.6)
**Primigravidae, n (%)**	17 (13.5)	68 (21.5)
**Multigravidae, n (%)**	109 (86.5)	248 (78.5)
**Education status, n (%)**		
- None	1 (0.8)	17 (5.3)
- Primary school	21 (16.7)	41 (13.0)
- Secondary school	97 (77.0)	210 (66.5)
- High school	7 (5.5)	48 (15.2)
**Marital status, n (%)**		
- Single	5 (4.0)	38 (12.0)
- Married	121 (96.0)	278 (88.0)
**IPTp-SP, n (%)**	104 (82.5)	268 (84.8)
**1 dose IPTp-SP, n (%)**	49 (47.1)	69 (25.7)
**≥2 doses IPTp-SP, n (%)**	55 (52.9)	199 (74.3)
**Bed net use, n (%)**	82 (65.1)	179 (56.6)

### ANC attendance and IPTp-SP and bed net use

Bed net coverage did not differ according to the total number of ANC visits (Table 2). IPTp-SP was offered to more than 80% of the population, and the first dose was taken usually between 16 to 28 weeks of pregnancy, though 14.8% of women received it during the 3^rd^ trimester (Table 1). Among women interviewed at term or delivery, 26.7% (118/442) had one dose of IPTp-SP (incomplete IPTp-SP), this concerned 39.2% of those with less than three ANC visits, and 21.8% of women from the group with complete attendance. The proportion of women receiving the full 2 or 3 doses of IPTp-SP was 57.5% (254/442), it increased with the number of ANC visits attended (Table 3). The number of SP doses was correlated with the number of ANC visits (Rho = 0.32, *p* < 0.01). A complete IPTp-SP course was more frequently administered to women with correct ANC attendance (63.0%) compared to the other group (43.7%).

**Table 3:** IPTp-SP and bed net use according to number of antenatal care visits

**Number of antenatal visits**						
	**1**	**2**	**3**	**4**	**5**	≥**6**
	**n = 15**	**n = 27**	**n = 84**	**n = 146**	**n = 95**	**n = 75**
**SP doses without bed net use, n (%)**						
0	4 (26.7)	4 (14.8)	2 (2.4)	11 (7.5)	9 (9.5)	7 (9.3)
1	2 (13.3)	2 (7.4)	9 (10.7)	13 (8.9)	9 (9.5)	10 (13.4)
2	0 (0.0)	3 (11.1)	18 (21.4)	31 (21.2)	23 (24.2)	20 (26.7)
3	0 (0.0)	0 (0.0)	0 (0.0)	3 (2.1)	0 (0.0)	1 (1.3)
**0 or 1 SP dose + bed net use**						
n, (%)	9 (60.0)	8 (29.7)	31 (36.9)	34 (23.3)	17 (17.8)	7 (9.3)
**≥2 SP doses + bed net use (correct prevention)**						
n, (%)	0 (0.0)	10 (37.0)	24 (28.6)	54 (37.0)	37 (39.0)	30 (40.0)

Among the 48 women with complete attendance who did not use any IPTp-SP, 27 (56.2%) said that they received SP tablets from the midwife and they did not want to take it, while the remaining women (n = 21) did not give any reason. Although correct ANC attendance was achieved in 71.5% of interviewed women, the proportion of those with correct malaria prevention (defined as at least two doses of SP associated with bed net use) was less than 50.0% (n = 155/316; 49.5%).

All the teenagers below 17 years old had at least three ANC and only two did not take IPTp-SP during their pregnancy; 63.2% (n = 12/19) of them received 2 doses, the same proportion slept under a bed net. Less than one third (n = 6/19; 31.6%) had a correct malaria prevention.

### Site to site health worker practice comparison

The ANC units of Melen and Owendo were compared according to the type of IPTp-SP administration by the midwives using the data from a subset of 157 women interviewed during their last antenatal care visit. At Owendo, SP was given orally to the women (n = 38) during ANC visit (supervised administration), whereas women attending in Melen (n = 119) took the drug at home (unsupervised administration). The majority of women from Owendo (n = 37; 97.4%) received a full course of IPTp-SP (≥ two doses), this was the case for only 64 (53.8%) of those interviewed at Melen. Similarly, 68% (n = 26/38) of women had a correct malaria prevention (IPTp ≥ two doses plus bed net) at Owendo, this concerned only 37% (n = 44/119) of pregnant women from Melen.

## Discussion

The WHO recommends at least four ANC visits, with three of them occurring after quickening, for pregnant women in order to receive and appropriate health care and timely intervention for malaria prevention strategies [2,5]. Our study confirms the high coverage of ANC attendance in Gabon, which ranged from one to eight visits. The WHO goal of four ANC visits was met by more than 70% of the interviewed women. This is considerable when compared to West and East Africa [9,15-17]. Improvement of strategies for pregnant woman care can explain this high attendance. Several community health centers and public hospitals, where health care services have been made affordable for the middle and low-income population, were built in the country these last five years. IPTp-SP is freely provided to the mothers and is always available in all the public health centers, even in the remote areas; and the cost of an ANC consultation is officially free of charge. Furthermore, each delivering woman must show an ANC card when arriving at the delivery unit they are distributed only during ANC. The timing of ANC was also found to be correct, with the majority of women having their first visit during the second trimester. Education sessions and oral communication from older women and mothers who encourage young pregnant women to go to ANC at three months of gestation may contribute to this. Moreover, after the first ANC, the midwife schedules the visit, reports the schedule in the ANC card and highlights each time the next appointment.

Regular attendance leads to the prevention, identification and treatment of maternal illnesses and obstetric complications. The Gabonese Ministry of Health recommends at least one dose of SP in the second trimester and one dose in the third trimester for malaria prevention, administered through ANC visits. Data from sentinel sites show acceptable adherence of midwives to these guidelines. IPTp-SP coverage is over 80%, it is considered high when compared to some countries where strategies have been adopted for more than 10 years [16-18]. Nevertheless, the goal of 80% coverage of two full course doses of SP has not yet been reached (57.4%), although the observed coverage is in range with reports from other settings [9,16-19]. In 2007, 56% of delivering women in the CHL received a correct chemoprophylaxis; it is noteworthy that this frequency has not increased over the last four years. The fact that almost 40% of delivering women with more than three ANC visits had no or partial SP uptake is a proof that several ANC visits are not sufficient to ensure complete IPTp coverage; barriers to complete IPTp coverage need to be explored. Indeed, comparison between on-site practices showed that when SP administration is supervised, coverage increases and ANC attendance is substantial.

Adolescent mothers from Sub-Saharan Africa are known to initiate ANC attendance late with a higher risk of low birth weight [20,21]. The present data suggest a better attendance for this population. The number of teenagers below the age of 17 years is too few to draw a conclusion and this study was not designed for this analysis. However, it would be interesting to investigate the behaviors of this subgroup several years after the previous study performed in Libreville and Lambaréné, and also after the implementation of new maternal and child health policy in the country [21].

This was not a survey evaluating knowledge and practices. An assessment of individual factors that may influence uptake of malaria prevention strategies revealed that high education level and higher gravidity did not predict complete attendance and good prevention. As previously stated, incomplete IPTp-SP may not be a problem deriving from pregnant women but a health center strengthening issue [9,21].

Bed net coverage is still low, below 60%. Although its utilization has been found to be associated with a reduced risk of malaria, it is difficult to assess its real coverage and impact. They are not always available in health centers where they are supposed to be distributed. ITNs distribution campaigns are organized from time to time in our country by the MNCP, there is no daily distribution to all pregnant women presenting at their first prenatal visit in all the health centers. Only one third (34%) of all the bed net used by the women are ITNs confirming the coverage model proposed by WHO [3].

Our study has some limitations. It was conducted in the capital city and surrounding areas, and may overestimate actual ANC visits and use of SP as attitudes and practices may differ from those in rural areas in terms of access to health care, health worker motivation and training, and availability of health service. Knowledge of malaria symptoms and prevention among pregnant women and health workers, as well as a correct evaluation of attitude and practice of midwives including lack of adherence to guidelines for offering IPTp-SP to all eligible women, were not performed in this survey. All these factors may influence the use of malaria preventive measures [18,22-25].

## Conclusion

Six years after the implementation of malaria control strategies according to the WHO in Gabon, this baseline survey performed at sentinel sites highlights that despite high complete ANC attendance, the use of full courses of IPTp-SP is still sub-optimal and insufficient. Evaluation and training of health workers, as well as surveys from other areas of the country are needed to further measure the impact of these strategies.

## Abbreviations

ANC: Antenatal care; CHL: Centre Hospitalier de Libreville; GMH: Gabonese Ministry of Health; IPTp: Intermittent preventive treatment during pregnancy; ITNs: Insecticide treated bed nets; MNCP: Malaria National Control Program; SP: Sulfadoxine-pyrimethamine; WHO: World Health Organization

## Competing interests

The authors declare that they have no competing interests.

## Authors’ contribution

The study was conceived and designed by MKBA; and supervised by MKBA, DPMM. MKBA, DPMM and MK interpreted the data. Data were analyzed and the manuscript was drafted by MKBA. MK advised on data analysis and reviewed the manuscript. All authors read and approved the final version.

## Pre-publication history

The pre-publication history for this paper can be accessed here:

http://www.biomedcentral.com/1471-2393/13/52/prepub
